# Awareness, Knowledge and Practice of Dental Professionals Regarding Biomedical Waste Management for a Green Dentistry: A Scoping Review

**DOI:** 10.3390/dj13120594

**Published:** 2025-12-11

**Authors:** Alice Murariu, Gabriela Luminița Gelețu, Livia Bobu, Simona Stoleriu, Gianina Iovan, Diana Zapodeanu, Bianca-Andreea Onofrei, Costin Iulian Lupu, Elena-Raluca Baciu

**Affiliations:** Grigore T. Popa University of Medicine and Pharmacy Iasi, Romania; alice.murariu@umfiasi.ro (A.M.); livia.bobu@umfiasi.ro (L.B.); simona.stoleriu@umfiasi.ro (S.S.); gianina.iovan@umfiasi.ro (G.I.); zapodeanu.diana@d.umfiasi.ro (D.Z.); bianca.onofrei@umfiasi.ro (B.-A.O.); iulian.lupu@umfiasi.ro (C.I.L.); elena.baciu@umfiasi.ro (E.-R.B.)

**Keywords:** waste management, dental practice, awareness, knowledge

## Abstract

**Background/Objectives**: Climate change is a major global issue affecting all facets of society, including dentistry. In response, the idea of green dentistry has developed, prioritising the reduction in environmental damage and the protection of patient health. This scoping review seeks to assess the level of awareness, understanding and practices of oral health professionals concerning the proper management of biomed. **Methods**: Searches were carried out in the Medline/PubMed, Scopus, Web of Science, Embase, and Google Scholar databases, analysing studies published between 2020 and 2025. Out of a total of 822 articles, 27 met the eligibility criteria. **Results**: In most of these studies, the respondents’ level of knowledge was found to be unsatisfactory or average, and only 17% of the studies reported respondents having a positive attitude towards adherence to sustainability principles. Although many professionals stated they were aware that dental waste could negatively impact the environment and human health if not properly managed, some still failed to provide correct answers to all the questions. **Conclusions**: The synthesis results indicated that oral health professionals have significant gaps in certain aspects of biomedical waste management, highlighting the need for proper training and to supplement the undergraduate and postgraduate curricula with courses on this topic.

## 1. Introduction

Climate change in recent years poses a significant challenge for all sectors, including healthcare, due to its severe meteorological impacts such as rising temperatures, changing rainfall patterns, floods, wildfires, and extreme droughts. All of these can disrupt the operation of healthcare facilities, including dental practices, through water shortages, power outages, damage to infrastructure, or interruptions in the supply of materials [1,2].

According to data provided by the World Health Organisation (WHO) in 2021, 3.6 billion people already live in areas highly vulnerable to climate change, which is expected to cause around 250,000 additional deaths each year in the future, solely due to malnutrition, malaria, diarrhoea, and heat stress [3].

Other adverse effects on humans include declining air quality, increased exposure to foodborne pathogens, inadequate nutrition, a rise in zoonotic diseases, and mental and respiratory health issues [3,4,5,6]. Dental activities also contribute to climate change through their carbon footprint, both from patient travel and the substantial amount of waste they produce [7,8].

To address these environmental concerns, the concept of green dentistry has been introduced as an eco-friendly approach that has gained significant attention in recent years. It aims to minimise the environmental impact of dental activities and to raise awareness among dentists about the negative effects [9,10,11,12,13]. Essentially, green dentistry refers to the practice of using technologies, procedures, and materials that do not harm the environment. This can be achieved through various actions, such as conserving water and energy, using non-hazardous products, reducing waste and eliminating harmful chemicals, as well as utilising eco-friendly dental materials [14,15,16].

In this context, biodegradable and biocompatible natural polymers (alginate, hyaluronic acid, starch, collagen, silk, fibrin, bacterial polyesters), along with synthetic polymers such as polylactic acid, poly (acrylic acid), poly (vinyl alcohol), and polyethylene glycol, are increasingly used in drug delivery, tissue engineering, stem-cell morphogenesis, wound healing, and regenerative medicine. They offer sustainable material options that align with the principles of green dentistry [17].

Currently, dental care professionals dispose of large amounts of single-use materials, plastics, packaging, and chemicals daily, all of which can harm the environment and public health [18]. For example, during the COVID-19 pandemic, a huge quantity of latex gloves and face masks was produced. These materials are not easily biodegradable but instead break down into tiny plastic particles [19]. Single-use materials make up a significant part of dental waste, which could be replaced with reusable alternatives [20].

By definition, biomedical waste (BMW) refers to waste generated during diagnosis, testing, treatment, research, or production of biological products [21]. According to data provided by the WHO, 85% of the total medical waste is considered non-hazardous, while the remaining 15% is hazardous because it is toxic, infectious, carcinogenic, flammable, corrosive, explosive, or radioactive [22]. Dental waste, in turn, is classified into biological waste, which includes materials contaminated with blood (cotton, gauze, masks); anatomical waste, such as extracted teeth or other by-products of surgical procedures; chemical and toxic waste, including mercury-based materials and disinfectants; sharps waste, such as scalpel blades, needles, and syringes; and pharmaceutical waste, such as anaesthetics [23,24].

According to the study published by Hackley [1], even ordinary traditional plastic toothbrushes take approximately 500 years to decompose. If replaced every three months, a person with an average lifespan would use about 300 toothbrushes over their lifetime.

The amount of waste generated from dental activities is substantial. For instance, in a study conducted in Turkey, Özbek and Sanin [25], after assessing the quantity of solid waste produced in dental practices across eight university clinics, found an average of 398.3 g/procedure/day. Another study carried out in Greece by Mandalidis and colleagues [26] reported an average of 53.3 g/patient/day in a study conducted in 20 dental clinics.

Proper segregation and disposal of waste are essential to reduce environmental impact and limit adverse effects on human health [27]. Poor management can pose serious risks to both health and the environment, including the transmission of infectious diseases, pollution, and physical injuries to workers and patients [28,29,30]. This concern has led international organisations in the field, such as the World Dental Federation (FDI), to propose a series of ethical and resource-efficient practices aimed at reducing pollution, lowering carbon emissions, and safeguarding future generations [31].

The prevalence of inadequate biomedical waste management practices varies among countries and is closely linked to the existence of national policies, guidelines, and recommendations regarding biomedical waste disposal. For example, a study from Brazil identified deficiencies in management activities; in Iran, 35.7% of dental centres dispose of dental waste in municipal bins without proper segregation or disinfection; and in Greece, substandard dental waste disposal practices are common in many dental clinics [24]. Countries with large populations, such as India and China, have extensive networks of dental clinics that produce increasing amounts of dental waste, including plastics, infectious materials, and toxic substances. This rising volume of waste presents significant challenges when proper segregation and disposal measures are not adopted. Implementing sustainable practices remains a notable challenge for private dental practices and hospitals, with marked differences observed between developed and developing nations. Barriers such as inadequate training for dental professionals, limited knowledge, lack of infrastructure, economic constraints, and the absence of suitable legislative frameworks all contribute to this issue [32].

As mentioned earlier, a particularly important aspect of sustainability in dentistry is the proper disposal of BMW, which requires a high level of knowledge among dentists, dental students, and other dental professionals regarding the management of waste generated from daily dental activities, specifically its sorting, collection, transport, and disposal, in accordance with legal regulations [33,34,35,36,37]. However, studies conducted on the knowledge, attitudes, and practices of oral healthcare personnel regarding dental waste have yielded varied results, making it difficult to obtain an overall picture that would help establish new future directions for effectively reducing the environmental impact of dental activities. In this context, the aim of this review is to evaluate the level of awareness, knowledge, and practice among dentists, dental students, and other dental professionals regarding the management of waste resulting from dental activities, by systematically mapping the findings of research conducted in this field over the last five years, and to identify any existing gaps in knowledge.

## 2. Materials and Methods

For the development of this scoping review, we followed the methodology proposed by Aromataris and colleagues in the JBI Manual of Evidence Synthesis [38], as well as the essential elements for conducting a scoping review outlined in the PRISMA extension for Scoping Reviews by Tricco [39], with the aim of ensuring a comprehensive and systematic approach (accessible through Supplementary Material Table S1). The research protocol was not registered in a public registry.

The key stages of the research include [40]:

1. Defining the research question;
2. Identifying relevant studies that match the defined research question;
3. Selecting studies using predetermined inclusion and exclusion criteria;
4. Creating the database;
5. Collecting and reporting the results.

### 2.1. Review Questions

The questions we sought answers to were as follows:

- Are dental professionals aware of the sustainability issues caused by current dental practices?
- What is their level of knowledge regarding the proper collection and disposal of waste generated in dental offices?

These questions were designed to guide the review process and achieve the objectives of this research.

### 2.2. Information Sources and Search Strategies

A systematic search was carried out using keywords in the PubMed, Scopus, Web of Science, Embase, and Google Scholar databases, covering the period from 2020 to 2025. The research strategy involved combining the keywords “green dentistry,” “awareness, knowledge, practice,” “biomedical waste management,” “sustainability,” and “eco-friendly dentistry” using Boolean operators such as AND and OR, to refine the search process. The aim was to retrieve all relevant articles from these databases. The search strategies are outlined in Table 1.

### 2.3. Inclusion and Exclusion Criteria

To ensure the quality and relevance of the articles included in this scoping review, a set of specific inclusion and exclusion criteria was established, as presented in Table 2. The inclusion criteria targeted full-text articles published in English between 2020 and 2025 that addressed sustainability in dental practice concerning medical waste, focusing on the awareness, knowledge, and attitudes of dentists, dental students, and other oral health professionals. The exclusion criteria eliminated articles published before 2020, abstracts only, or those written in languages other than English, as well as non-free or restricted-access articles (not accessible through the university), duplicates, and studies referring to other categories of healthcare professionals. Additionally, systematic reviews, narrative reviews, scoping reviews, and irrelevant studies that did not align with the stated objective were excluded.

The article selection process was conducted in two stages. In the first stage, titles and abstracts of the identified articles were screened for relevance based on the inclusion and exclusion criteria. Next, the full texts of the available articles were independently reviewed by three reviewers (A.M., S.S., and L.B.) to confirm they met the inclusion and exclusion criteria and to identify potentially eligible studies. Inter-reviewer agreement was assessed using Cohen’s kappa statistic, which showed excellent agreement (κ > 0.80). Any disagreements between reviewers were resolved through discussion until consensus was reached or through consultation with an additional evaluator (E.-R.B.).

To ensure a comprehensive database, data extracted from the selected articles were recorded in a specially designed form, which included the first author and publication year, study design, study objective, population, the assessment instrument used, and key findings and conclusions (Table 3).

To address the research questions and objectives, the studies were categorised and data were summarised, including the year of publication, study area, study design, group of professionals, and findings. By synthesising the collected data, the study aimed to provide a clearer understanding of the participants’ level of knowledge, highlight any gaps or uncertainties in the current research, and outline possible directions for future studies.

## 3. Results

Between July 2020 and September 2025, a total of 822 articles were identified and retrieved from the following databases: PubMed/MEDLINE (134 articles), Scopus (313), Web of Science (150), Embase (128), and Google Scholar (97). Of these, 217 were excluded because they were either duplicates, abstracts only, review articles, outside the analysed period, or not open/institutionally accessible. From the remaining 605 records, 329 were found to be irrelevant, leaving 276 for eligibility assessment.

Of these, 249 articles were excluded for reasons such as lack of relevance to the topic, not focusing on the subject, not addressing waste management, not involving dental professionals, or lacking a proper methodology. Finally, 27 articles were included in the analysis.

**Figure 1:**
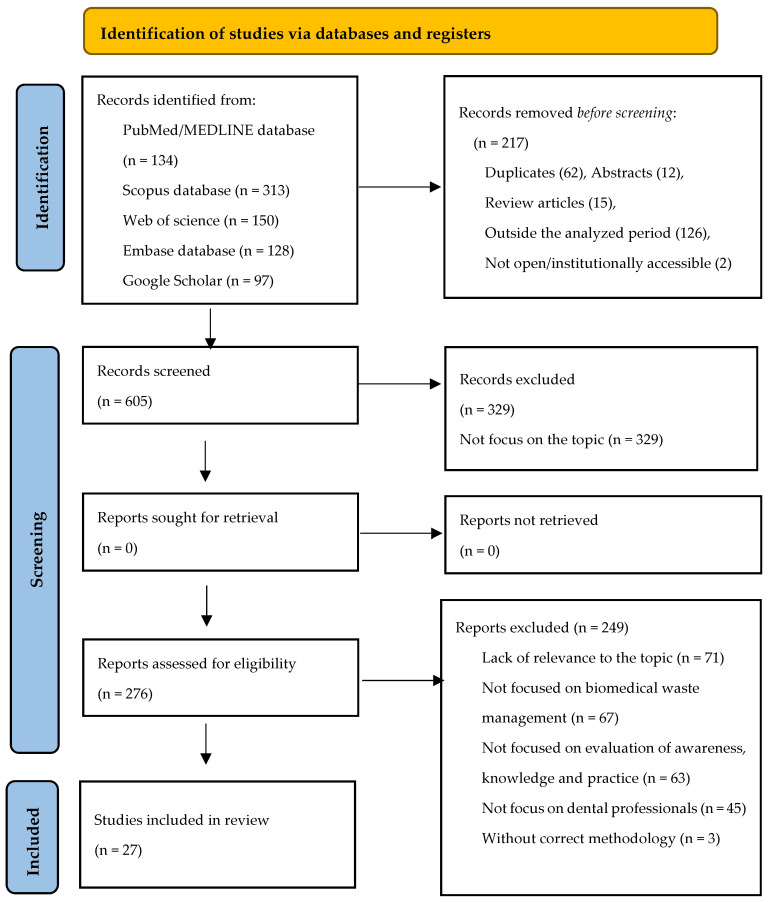
The flow diagram for the identification, screening and eligibility of studies (PRISMA-Scr).

The search results and study inclusion process are presented in a PRISMA-ScR (Preferred Reporting Items for Systematic Reviews and Meta-Analyses extension for Scoping Reviews) flow diagram in Figure 1 [39].

### General Characteristics of the Selected Articles

**Figure 2:**
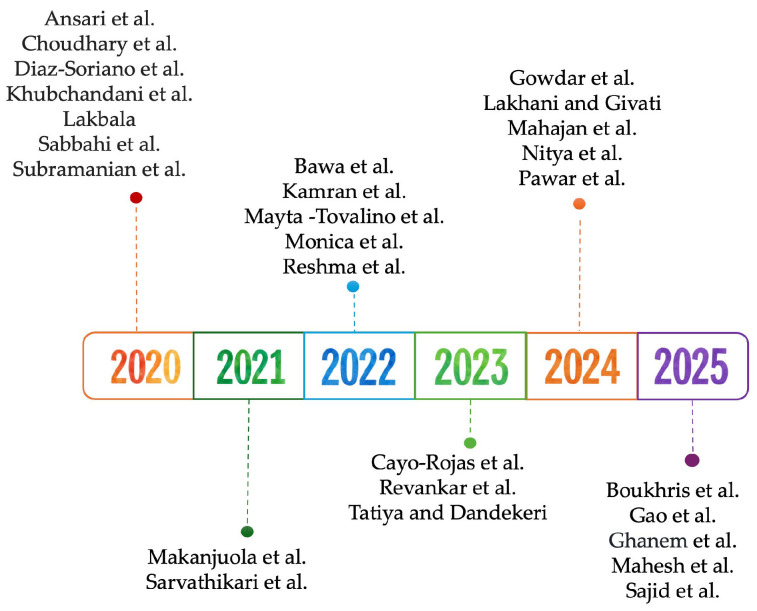
Timeline of included studies [41,42,43,44,45,46,47,48,49,50,51,52,53,54,55,56,57,58,59,60,61,62,63,64,65,66,67].

Following the analysis, a total of 27 studies published between 2020 and 2025 were identified. The highest number of studies were published in 2020 (7), followed by 2022, 2024, and 2025, with five articles each, three articles in 2023, and two articles in 2021 (Figure 2).

**Figure 3:**
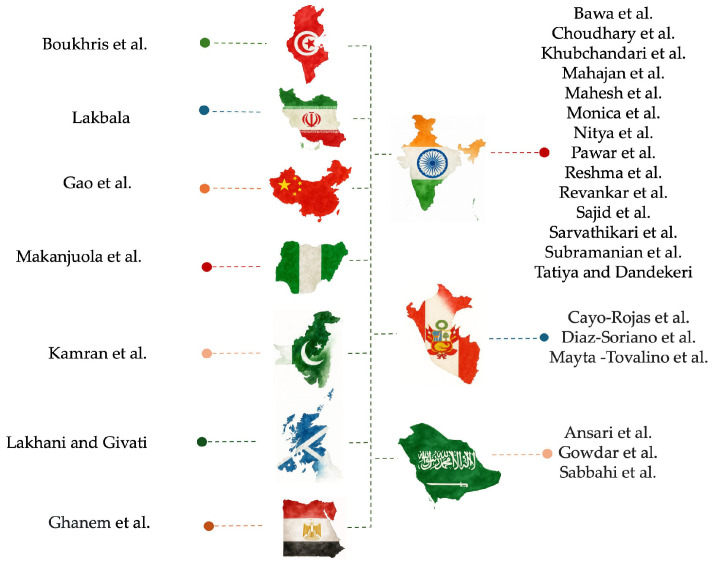
Mapping authors to countries in the dataset [41,42,43,44,45,46,47,48,49,50,51,52,53,54,55,56,57,58,59,60,61,62,63,64,65,66,67].

Among the analysed research, 24 were cross-sectional surveys, one was a quasi-experimental pre/post study, one was a prospective longitudinal study, and one was a qualitative study based on the Theory of Planned Behaviour model. The studies were conducted in various countries, with India contributing the largest number of articles (14), followed by Peru (3), Saudi Arabia (3), and one article each from China, Egypt, Iran, Pakistan, Nigeria, Tunisia, and the United Kingdom—Scotland (Figure 3).

Out of the 27 articles analysed, 10 focused on dental students, while the remaining studies targeted a wider group of professionals, including dentists, residents, interns, dental assistants, and academic staff from dental faculties.

The data collected from the included articles are detailed in Table 4 and organised according to the following parameters: author and year, study population, sample size, sample area, study type and instrument, findings, and conclusions.

This synthesis of studies conducted across various countries shows that dental professionals—students, interns, practitioners, nurses, and faculty members—generally exhibit insufficient knowledge, inconsistent attitudes, and poor practices regarding biomedical waste management. Although awareness of the importance of BMW segregation is relatively high, a significant proportion of participants were unfamiliar with regulations, colour-coding protocols, and proper disposal methods for materials such as amalgam, sharps, and chemicals. Training deficiencies were consistently noted; despite some awareness, a lack of structured instruction often led to poor adherence to established procedures. Several studies found that targeted educational interventions notably enhanced knowledge, attitudes, and behaviours, particularly among students. However, systemic barriers—such as inadequate infrastructure, infrequent waste collection, and unclear guidelines—also obstruct effective implementation. Consequently, most studies highlight the urgent need for standardised training at all professional levels, ongoing education programmes, and institutional support to ensure compliance and foster sustainable waste management in dental settings.

## 4. Discussion

Green dentistry involves using sustainable materials and techniques, along with environmentally friendly approaches to waste management, energy conservation, patient care, and educational initiatives. It also encourages waste management procedures that ensure proper sorting, collection, storage, transportation, treatment, and disposal of dental waste in accordance with local regulations [68].

Sustainability in dentistry represents a moral and ethical duty for every dentist to adopt responsible practices based on the 4R principle: Reduce, Reuse, Recycle, and Rethink [69,70]. This approach aims to create a positive environmental impact through actions such as reducing material consumption via reuse, responsible waste management, recycling dental instruments and materials, minimising water and energy use, promoting eco-friendly transportation for both dentists and patients, and expanding the use of preventive and digital techniques (digital radiography, CAD/CAM systems), as well as artificial intelligence and teledentistry to lower unnecessary travel to dental clinics [71,72,73,74,75].

Currently, many international organisations have launched initiatives to protect the environment and support eco-friendly dental practices. For example, the World Dental Federation (FDI) adopted a statement on sustainability in dentistry in 2017 and issued guidelines for oral health professionals. These guidelines encourage reducing the use of energy, water, paper, and environmentally harmful materials, as well as minimising air emissions and wastewater discharge, representing a strong call to raise awareness about these issues [31].

All concerns related to the environment and public health cannot be effectively addressed without genuine awareness from dentists, students, the dental team, and decision-makers within university leadership, who have a moral obligation to incorporate courses on sustainability and green dentistry into the university curriculum [76,77,78,79,80,81].

Based on the review of the selected articles, common themes include mercury management, handling of radiographic developing solutions and gypsum, segregation and disposal of BMW in colour-coded containers, management of sharp objects, and educational interventions related to ecological dentistry.

### 4.1. Management of Hazardous Waste: Mercury and Silver Ions

The gradual removal of dental amalgam is a decision mandated and implemented through the Minamata Convention, drafted by the United Nations Environment Programme in October 2013 and signed by over 100 countries [82,83,84].

In the European Union, dental amalgam has been progressively phased out by 1 January 2025, with the production and import of dental amalgam also to be gradually eliminated by 30 June 2026 [85].

As a signatory to the Minamata Convention, China is committed to progressively reducing the use of dental amalgam. A similar situation exists in the United States, where the use of dental amalgam remains permitted in practice [86].

Mercury from dental amalgam fillings poses a threat to both the environment and human health. Despite all safety measures in dental practice, mercury is released during the placement and removal of amalgam restorations, posing a direct risk to patients and dental professionals alike [87].

This legislation applies only within the European Union, as countries such as Nigeria, Saudi Arabia, Peru, and India still routinely use amalgam fillings in dental procedures, despite being signatories to the Minamata Convention [56,57,63,65].

In Nigeria, 85.7% of participants acknowledged placing amalgam fillings, while 11.7% were unaware of the harmful effects of mercury. Most amalgam waste is disposed of in regular trash bins or via sewage systems [57].

Studies involving dental students revealed that many lack adequate knowledge of proper disposal methods for dental amalgam. In Peru, Mayto-Tovalino [56] reported that 71% of female students and 28.9% of male students discarded excess amalgam into regular trash bins; meanwhile, in Saudi Arabia, only 8% of students used this incorrect method [63].

Another study by Khubchandani found that amalgam fillings are still commonly used in Indian dental schools, with only 27% of students appropriately disposing of excess mercury and mercury-contaminated gauze in sealed containers [51]. Similarly, Mahesh reported that 48% of all participants—including faculty, undergraduate, and postgraduate students—had knowledge of proper disposal procedures for mercury [55].

Amalgam fillings are also used in Egyptian dental practice, with Ghanem’s study showing that only 38% of practitioners store amalgam in sealed containers, while recycling is rarely performed [48]. The same trend is seen in Iran, where only 27.1% of dentists utilise amalgam separators [52].

Other hazardous wastes in dental clinics include fixer and developer solutions from radiology units, along with undeveloped X-ray films, which contain significant amounts of silver ions that can reach water systems if not disposed of properly [25,66,70].

In India, Bawa [42] reported that only a small percentage (18.22%) of dental students follow the correct disposal methods for biomedical waste, while in Peru, 54.7% of students were unaware of which components of dental amalgam are environmentally harmful [42,46]. Similar patterns are observed in studies from Iran and India [52,54].

### 4.2. Other Dental Waste

Among the dental materials analysed, apart from amalgam and silver ions, only gypsum was included in the questionnaire items. Although impression materials and gypsum-based products are not hazardous when dry, they become dangerous upon water absorption, as they can emit harmful microorganisms and gases such as hydrogen sulphide.

In India, Subramanian found that 62.75% of students, 54.8% of practitioners, and 76.9% of faculty members considered impression materials to be hazardous and disposed of them in yellow bags [66].

### 4.3. Segregation of Biomedical Waste into Colour-Coded Containers

In the dental office, a significant amount of waste is produced, including plastic, latex, cotton, glass, amalgam waste, disinfectants, chemicals, dental impression materials, surgical needles, extracted teeth, blades, and human tissues. All these materials are hazardous because they are contaminated with saliva and blood, which may carry pathogenic microorganisms. Non-hazardous waste comprises single-use paper towels, packaging materials, and surface coverings [71].

Colour coding of biomedical waste containers is crucial as it minimises the risk of contamination and injury to healthcare staff and aids in the separation of medical waste [59]. In this context, each country has clear regulations on colour standardisation and labelling of medical waste, and dental practitioners should be familiar with the legislation and adhere to the established regulations.

Once waste is separated, dentists should use appropriate containers for each waste category. Sharp objects, such as scalpel blades or needles, must be disposed of in puncture-resistant containers to prevent contamination or injury during handling and transport. Contaminated materials, like gloves and gauze, should be collected in sealed bags to avoid potential exposure to infectious agents. Chemical waste must be stored in tightly closed containers with proper labels, ensuring disposal in accordance with local regulations [88].

Although the coding of medical waste may vary between countries, the following colour codes should generally be followed: yellow for most infectious waste, anatomical waste, extracted teeth, blood-soaked materials, and certain chemical and pharmaceutical waste; red for biohazardous materials; white for sharps (needles, blades), which must be disposed of in leak-proof, puncture-resistant containers; blue for broken or discarded glassware; black for general non-hazardous waste (packaging, daily waste) [55].

An analysis of the reviewed articles reveals a general trend regarding the knowledge of dentists and dental students on this subject: although most are aware of the importance of disposing of waste in separate containers, their correct responses to questions about specific types of waste varied considerably.

In Saudi Arabia, Gowdar [49] reported high percentages of correct answers concerning the disposal of waste in colour-coded containers, ranging from 70% to 79% among dental students and from 78% to 93% among practising dentists. However, another study conducted in Saudi Arabia found much lower figures, with only 67% of dentists being aware of these practices [63]. In Pakistan, 64.4% of practitioners were unaware of BMW management regulations [50], while in Iran, dentists exhibited inadequate knowledge: the main method for disposing of X-ray film lead foils (52.6%), orthodontic wires (48.9%), and expired drugs (60.2%) was the trash bin. Chemicals used in dentistry and amalgam waste were predominantly poured into the municipal sewage system [52].

Boukris [43], in a study involving 50 dental clinics in Tunisia, found that only 70% of clinics practised selective segregation of BMW. The efficiency of waste recycling was 60%, with a contamination rate of 20% due to mixing BMW with recyclable waste.

In India, several studies present differing results: Nitya [59] reported 63–79% correct answers to the same set of questions; Sarvathikari [65] found a correct answer percentage of 58.7%; Mahesh [55] recorded values ranging from 61.7% to 40.6% for questions about the disposal of blood-contaminated waste in yellow bags and pharmaceutical waste in black bags; in a study by Choudhary [45], correct responses to questions on legislation and colour-coded segregation of medical waste varied from 33% among students, 62.2% among residents, and 100% among medical staff. Monica [58] in India discovered that only 55.9% of practitioners were aware of the colour coding for infectious waste (extracted teeth).

Regarding Indian students, Sajid [64] found that 69.3% did not possess satisfactory knowledge, in contrast with Revankar [60], who reported that 70.4% of students provided correct answers. Similarly, in Peru, Mayto-Tovalino [56] observed that dental students were familiar with the concept of BMW segregation, with a higher proportion among females (70.3%) compared to males (29.6%) (*p* = 0.004).

By contrast, in Nigeria, Makanjuolo [57] found a very high percentage of practitioners (82.8%) who had poor knowledge regarding amalgam management and dental waste legislation.

Another aspect identified in many of the analysed articles refers to the discrepancies between theoretical knowledge and practical results concerning the BMW colour-coding system. For instance, Ghanem [48] in Egypt observed that 84% of respondents were familiar with the coding system, yet 77% scored poorly on the knowledge assessment. In the study by Boukris [43], 68% of participants demonstrated basic understanding of waste segregation, but only 42% adhered to consistent waste management protocols. In India, 58% of respondents knew that segregation is carried out based on colour codes, yet 26.5% provided incorrect answers.

Many countries face significant challenges in managing medical waste. In China, for example, the healthcare sector produces around 650,000 tonnes of medical waste each year, with the volume rising by approximately 20% annually in both urban and rural areas. The Chinese government addresses this issue through Law 380 and Regulation 287, which classify hazardous medical waste into five types: infectious, pathological, sharps, general medical, and chemical waste. Despite these regulations, the effective management of medical waste remains inconsistent nationwide. As a signatory to the Minamata Convention, China is required to gradually reduce the use of dental amalgam. A similar situation exists in the United States, where the use of dental amalgam continues to be allowed in practice [89].

### 4.4. Disposal of Sharp Waste

Sharps containers are specifically designed for the collection and disposal of sharp objects such as needles, syringes with attached needles, scalpel blades, clinical glassware, or other items that can cause cuts or punctures. Sharp objects must be placed in a puncture-resistant container designed specifically for the safe management of sharps.

From the studies analysed, it was found that only 50% of students in India, in the study published by Khubchandani [51], and 50% of those in Saudi Arabia, as reported by Ansari [41], follow the proper protocol for disposing of syringes and other sharp objects.

A higher percentage was observed by Mahesh [55] among Indian students—73.8%—while in Peru, 58.6% of female dental students and 41.3% of male students were aware of the appropriate disposal methods for contaminated needles [56]. In contrast to these results, the study conducted by Mahajan [54] in India reported that only 37.86% of students attained a satisfactory level of knowledge. Regarding other professional groups, Pawar [60] found in India that 70% of dentists and 60% of dental assistants possessed knowledge about the disposal of sharps, in contrast to Reshma’s study, also carried out in India, which reported a significantly lower percentage—only 30.4% [61].

### 4.5. Educational Interventions in Ecological Dentistry

The analysis of the articles reveals a lack of comprehensive understanding regarding the proper management of hazardous waste generated in dental practices. However, the findings reported by Bawa in India show that 70.3% of students did not wish to receive further information, in contrast to 89% of Egyptian students who believed they required more education on the subject [42,48].

Two articles highlighted the importance of educational interventions as vital measures for increasing awareness and ensuring the correct application of waste disposal rules in daily dental practice.

The first study was conducted in China by Gao [47], demonstrating that an educational intervention can significantly enhance the knowledge, attitudes, and practices of professionals (dentists, students, and nurses) concerning medical waste management. The authors concluded that the greatest improvements in knowledge and practice were observed among nurses and dental students. Even experienced dentists, who initially had high knowledge scores, benefited from the training and the updating of waste management protocols.

The second study was published by Cayo-Rojas [44] in Peru, assessing the knowledge of 165 dentists regarding BMW management at three time points: before the intervention, immediately after, and 14 days post-intervention. The intervention consisted of a 9.27 min YouTube video presentation about the recycling, reuse, and proper disposal of dental materials, as well as the correct management of hazardous waste. The authors concluded that the dentists exhibited higher knowledge scores immediately after the presentation; however, after 14 days, some of them, particularly those with less than 10 years of experience, did not retain the same high level of knowledge.

To update information and shift attitudes towards BMW management, Lakhani and Givati in Scotland [53] demonstrated through a qualitative study based on interviews with 15 professionals, using the Theory of Planned Behaviour model, that this approach can effectively support behaviour change.

After analysing the selected articles, we can address the initial questions regarding the level of awareness, knowledge, and practice among oral health professionals: although many are aware of the importance of environmental factors in their daily work, the overall level of knowledge remains unsatisfactory or moderate for most participants, as reported in 83% of the studies analysed. The remaining 17% of the articles indicated that certain categories of professionals, such as dental students, display a positive attitude towards sustainability in dentistry.

### 4.6. Incorrect Practices, Regulatory Gaps and Inadequate Training

From the analysis of the selected articles, the following negative aspects regarding the proper management of BMW are observed:

- Incorrect practices in Tunisia and India concerning waste incineration procedures [43,66];
- Improper disposal of amalgam in general waste bins, as well as the absence of amalgam separators in Saudi Arabia, Iran, Peru, and Nigeria [41,52,57,65];
- Disposal of toxic liquids and chemicals into the public system in Iran and Nigeria [52,57];
- Incorrect segregation of BMW by colour in Tunisia, Saudi Arabia, India, Iran, and Nigeria [43,49,51,52,57].

Some authors have highlighted issues that stem from insufficient or outdated legislative regulations in countries such as Saudi Arabia and India [49,67], inadequate infrastructure in India and Tunisia [43,67], or other barriers that hinder the proper management of BMW, as specified by Sarvatikari: excessively large bins, infrequent waste collection, and additional expenses [65]. Lastly, many authors have reported the lack or inadequate training on this topic, including Reshma [61] in India, Kamran [50] in Pakistan, Ghanem [48] in Egypt, and Sabbahi [63] in Saudi Arabia.

### 4.7. Heterogeneity of Selected Studies

The study results show significant variations in the number of correct responses, possibly due to the diverse group of professionals participating, who differed by profession (dentist, student, resident, nurse, or academic staff), country or region, experience, and specialisation.

Besides the variability among participants, there are also differences in the quality of the questionnaires used in each study, which vary in terms of the number of items, the scoring system, or sometimes the topic addressed, making the results difficult to compare. Other possible reasons for this variability could include legislative gaps related to medical waste management, the possibility that such regulations are not accessible to all practitioners, and the limited or absent training courses or sessions on this subject.

Another aspect that should be considered is the challenges and barriers faced in implementing eco-friendly procedures and materials, which may discourage many dental clinics with limited financial resources, insufficient human resources, or low interest in this topic [90,91]. Additionally, dental schools should take an active role by promoting the ecological aspects of dental practice within their educational curricula.

### 4.8. Gaps in Literature and Future Research Challenges

Further research is necessary to gain a clearer understanding of this subject, by examining other categories of dental materials with potential toxicity to the environment but commonly used in dental practice, such as composite resins, alginates, methyl methacrylate, and disinfectant solutions.

### 4.9. Clinical Implications

The practical value of this scoping review extends to dental practitioners and students, who can be encouraged in their daily practice to adopt innovative, environmentally friendly technologies, properly dispose of medical waste, and recycle certain old instruments and non-hazardous materials from the dental office, such as plastic and paper.

### 4.10. Study Limitations

Despite the high methodological standards maintained, this study has several limitations. Some articles may have been overlooked, although our search criteria aimed to be as comprehensive as possible. Only articles published between 2020 and 2025 and written in English were included, and studies lacking a clear research methodology were excluded. Following PRISMA ScR and JBI guidelines, a formal quality or bias risk assessment was not performed, as this review’s primary goal was to map the current literature rather than evaluate evidence strength or perform a restricted analysis. However, this may affect the relevance of the findings.

Another limitation is that most of the included studies originate from India, which limits the ability to generalise the results on a global scale.

## 5. Conclusions

In light of the findings of this scoping review, the following conclusions can be drawn:

- Implementing sustainability in dental practice is both a current necessity and a moral obligation. This scoping review highlights significant gaps in awareness, knowledge, and professional practice regarding the proper management of biomedical waste (BMW).
- Although some studies reported a generally positive attitude among dental professionals, even these individuals showed the need for further updating and improvement in their knowledge.
- The shift towards sustainable dental care is no longer optional; it has become essential in response to ongoing environmental, economic, and public health challenges, including those highlighted by the COVID-19 pandemic.
- Each country must establish clear and accessible protocols and guidelines for the proper segregation of BMW, provide support and opportunities for dental practices to adopt sustainable measures, and actively promote awareness among healthcare professionals.

## Figures and Tables

**Table 1:** Search strategies.

Database Name	Search Terms	Number of Articles Found
PubMed/Medline	green dentistry OR sustainability dentistry AND waste management, awareness, knowledge, practice OR sustainability AND dentists AND dental students AND biomedical waste management	134
Scopus	awareness AND knowledge AND practice AND green dentistry AND waste AND management OR sustainability AND dentists AND dental students AND dental practice AND knowledge AND biomedical waste	313
Web of science	green dentistry AND waste management OR biomedical waste AND awareness AND knowledge AND dental practice OR sustainability AND dentistry AND waste management AND awareness OR eco-friendly dentistry AND waste management AND practice AND dentists AND dental students	150
Embase	waste management AND dental practice OR awareness, knowledge, practice AND waste management AND green dentistry OR sustainability dentistry AND waste management OR eco-friendly dentistry AND biomedical waste AND knowledge	128
Google Scholar	biomedical waste management, dentists, dental students OR green dentistry, awareness, knowledge, dental practice, medical waste OR eco-friendly dentistry, medical waste, knowledge, sustainability dentistry, awareness	97

**Table 2:** Inclusion and exclusion criteria.

Criterion	Inclusion	Exclusion
Type of study	Cross-sectional studies, analytical studies, qualitative studies	Systematic review, narrative review, scoping review
Participants	Dentists and dental students	Professionals from other healthcare specialties, staff from other medical and educational institutions (physiotherapists, general medicine nurses, economists)
Evaluation period	Articles published between 2020–2025	Articles published before 2020
Disponibility	Full-text articles in English, with open access or access through the university	Articles in abstract or not freely accessible, articles published in other languages than English
Intervention	Studies that aim to evaluate the level of awareness, knowledge and practice regarding waste in the dental office	Irrelevant articles, which do not correspond to the proposed purpose, or which do not have a clear research methodology

**Table 3:** Data extraction template.

Category	Details
General Information	The first author of the article, year of publication, country and region where the research was conducted
Study design	Cross-sectional studies/analytical studies
Study objective and aim	The main objectives of the research: to evaluate the level of awareness, knowledge and practice regarding the disposal of infectious and toxic dental waste from the dental office
Population	Sampling method, number of subjects, age, type of professionals, dental specialisation, experience
Intervention	Type of questionnaire used, number of items, validation; other types of interventions
Key findings and conclusions	Main findings, conclusions, future research suggestions, limitations

**Table 4:** Characteristics of selected study.

Author, Year	Study Population	Sample Size	Sample Area	Study Type and Instrument	Findings	Conclusions
Ansari et al., 2020 [41]	Dentists	315	Saudi Arabia	Cross-sectional study; Questionnaire with 16 items	75% of dentists were aware of the necessity of waste segregation and of Occupational Safety and Health Act (OSHA) guidelines. In dental practice, 41% of the participants reported incorrect disposal of amalgam and half of them—of sharp instruments. Male participants presented better knowledge and attitude regarding BMW disposal and OSHA guidelines.	Dental practitioners and dental students should be trained for BMW management by attending specialised programmes.
Bawa et al., 2022 [42]	Undergraduate students in dental surgery and interns	845	India	Cross-sectional study; Questionnaire with 24 items	Almost half of the undergraduate students in the third and sixth year of study had good knowledge on BMW and had a positive attitude regarding the rules for waste management (47% and 48%, respectively). Third-year students had significantly better knowledge and more positive attitude when compared to the students in the sixth year of study and the interns.	Very few participants (34%) followed the rules regarding BMW management in a correct manner. Education of dental students (good knowledge and correct practice) regarding BMW management is needed.
Boukhris et al., 2025 [43]	Dental professionals	120	Tunisia	Cross-sectional study; A structured questionnaire consisting of 30 questions	While 68% of participants understood waste segregation, only 42% consistently followed proper procedures. Key barriers included a lack of training (with just 30% receiving formal instruction) and inadequate infrastructure (44% lacked proper bins). Half the clinics still used incineration despite its environmental impact.	Addressing operational, financial, and knowledge-related barriers may lead to improvements in waste segregation, recycling, and disposal practices among dental professionals.
Cayo-Rojas et al., 2023 [44]	Dentists	165	Peru	Longitudinal prospective study assessing the effects of a virtual educational programme on the knowledge and awareness of dental material recycling and reuse, as well as BMW management; A 30-item questionnaire	Recycling, dental material reuse, and biomedical waste management response rates improved post-test (*p* < 0.05), but after 14 days, knowledge retention remained low among private university participants, unmarried individuals, bachelor’s degree holders, non-specialists, non-teachers, and those with under ten years’ experience.	Government agencies should support oral health professionals researching educational methods to improve and assess the sustainability and environmental impact of dental practices. These efforts help advance knowledge and enable effective evaluation of dentistry’s environmental footprint.
Choudhary et al., 2020 [45]	Undergraduate students, residents, and nursing staff	95	India	Cross-sectional study; Questionnaire with 10 items	The highest score of correct answers was recorded by nursing staff and the lowest by undergraduate students.	More awareness of the dentists is needed to improve BMW management. The topic should be included in the university curriculum of undergraduate students and continuing education programmes should be conducted.
Diaz-Soriano et al., 2020 [46]	Dental students of Public University Peru	254	Peru	Cross-sectional study; The questionnaire used is Biomedical Waste Management Awareness & Knowledge with 30 items	The aim of the study was to examine the influence of age, gender, year of study, and marital status on the level of knowledge and awareness about biomedical waste using logistic regression analysis. The results showed that none of the analysed variables influenced the level of knowledge: age (OR = 0.96; CI: 0.85–1.08), gender (OR = 1.69; CI: 0.98–2.90), year of study (OR = 1.18; CI: 0.91–1.54), marital status (OR = 1.84; CI: 0.14–23.68).	The authors concluded that students are aware of and knowledgeable about the management and recycling of BMW from dental products.
Gao et al., 2025 [47]	Dental professionals: dentists, dental nurses, dental students	187	China	Cross-sectional quasi-experimental study; Questionnaire with 30 items	The study examined the level of knowledge, attitudes, and practices of dental professionals at two time points: before and after the BMW training programme. Post-intervention, knowledge, attitude, and practice scores rose by 22.3, 17.1, and 22 points, respectively. Students showed the highest gains, with statistically significant differences compared to dentists (*p* = 0.002 for knowledge, *p* = 0.001 for attitude, *p* = 0.004 for BMW practice).	The authors demonstrate the important role of educational interventions that can substantially improve dental professionals’ knowledge, attitudes, and practices regarding BMW management.
Ghanem et al., 2025 [48]	Dentists, dental students, and interns	257	Egypt	Cross-sectional study; Questionnaire with 37 items	Less than half of participants (40.1%) knew about BMW laws and rules in Egypt and about 77% of them had poor knowledge regarding BMW management. An increased percentage (84%) disposed of the waste in different coloured bags. Even half the participants were trained in dental waste management, and an increased percentage (89.1%) considered that more training is needed.	Dentists and dental students presented considerable lack of knowledge and low laxity in performing BMW management. Knowledge level was significantly associated with the education level.
Gowdar et al., 2024 [49]	Dental students and dental practitioners	200	Saudi Arabia	Cross-sectional study; Questionnaire with 20 items	A high percentage of dental practitioners and dental students were aware of different categories of BMW (72% and 56%, respectively), of the law and rules of BMW management (72% and 86%, respectively), and had knowledge about colour coding for waste segregation (79% and 48%, respectively). Dental practitioners had slightly more awareness and knowledge regarding BMW disposal when compared to dental students.	Better knowledge regarding BMW management of dental practitioners was reported when compared to dental students.
Kamran et al., 2022 [50]	Undergraduate and postgraduate dental students, and dental practitioners	273	Pakistan	Cross-sectional study; Close-ended questionnaire with 25 items	64.4% of respondents were unaware of dental waste regulations, but 67.7% knew waste categories. Most (95.5%) supported training, with postgraduates scoring highest (*p* = 0.069).	The overall knowledge regarding environmentally friendly waste management was found to be inadequate among dental students and practitioners.
Khubchandani et al., 2020 [51]	Dental undergraduate students, interns and postgraduate students	168	India	Cross-sectional study; Questionnaire with 45 items	High percentages of participants had no training on biomedical waste management and were not aware of legislations (80% and 70%, respectively). Also, 70% considered that they do not have adequate knowledge regarding BMW management. A small percentage (10%) did not practice waste segregation on specific categories.	The knowledge and practice regarding BMW management should be improved, despite the positive attitude and the awareness of dental practitioners and students.
Lakbala, 2020 [52]	Dentists	133	Iran	Cross-sectional study; Self-administered questionnaire with 40 items	Only 12.0% of dentists knew how to use the yellow container for human anatomical waste. Most disposed of x-ray film lead foils (52.6%), orthodontic wires (48.9%), and expired medications (60.2%) in the trash, while chemicals and amalgams were typically poured into municipal sewage.	Overall, dentists demonstrated limited awareness regarding dental waste management, and their waste disposal practices often did not meet established standards. Consequently, there is a need to implement a comprehensive healthcare waste management system in Iran.
Lakhani and Givati, 2024 [53]	Dental professionals (dentists, dental nurses, dental hygienists)	15	Scotland	Qualitative study; Semi-structured interview of 20–30 min; Responses were analysed using the Theory of Planned Behaviour framework.	Thematic analysis identified eight themes related to participants’ attitudes, subjective norms, and behavioural control, highlighting that knowledge gaps and lack of awareness often corresponded with low intent to engage in sustainable waste management.	The study highlights that using broader behaviour change theories and models is crucial for developing effective interventions to encourage sustainable waste segregation among health professionals.
Mahajan et al., 2024 [54]	Dental undergraduate students	450	India	Cross-sectional study; Questionnaire with 37 items	A very low percentage of undergraduate dental students had excellent level of knowledge concerning the legislation and the practice of BMW management (10.19% and 6%, respectively). Almost 30% of the participants showed a poor attitude regarding proper BMW management.	Undergraduate dental students did not have adequate knowledge, awareness, and attitude regarding the adequate practice of BMW management.
Mahesh et al., 2025 [55]	Undergraduate and postgraduate students from a dental college, and faculty members	180	India	Cross-sectional study; Questionnaire with 59 items	41.7% of respondents knew black is for general waste, while 73.8% correctly identified puncture-resistant bins for needle syringes. Mercury disposal awareness was 42.8%. 53.9% were aware of sharps containers and blood waste incineration. Faculty members scored highest overall.	The study highlights the importance of emphasising biomedical waste training, especially for undergraduate students, to promote uniform compliance with safety and disposal protocols within healthcare environments.
Mayta-Tovalino et al., 2022 [56]	Dental students from the Faculty of Dentistry of the Universidad Nacional Mayor de San Marcos	254	Lima, Peru	Cross-sectional study; Questionnaire with 13 items	70.3% of female students knew about separating BMW into separate containers, compared with 29.6% of male students; 71% of female students and 28.9% of male students disposed of excess amalgam in the common trash bin, while 49.4% of female students and 50.5% of male students discarded lead foils in the common trash bin.	The logistic regression model showed that age, gender, year of study, and marital status did not significantly influence the level of awareness, knowledge, attitude, or practices regarding BMW management.
Makanjuola et al., 2021 [57]	Dentists, dental students of 5th and 6th years, dental nurses, dental therapists	437	Nigeria, Lagos State	Cross-sectional study; Self-administered questionnaire with 31 items	95.9% had poor medical waste management practices; 42.3% disposed of hazardous liquid waste into the sewage system; none of the respondents used an amalgam separator. There were statistically significant differences in BMW practices according to years of experience (*p* = 0.01), student/dentist status (*p* = 0.006), and place of work (*p* = 0.006).	82.8% of respondents had insufficient knowledge regarding dental amalgam management and BMW legislation.
Monica et al., 2022 [58]	Orthodontists and General dental practitioners	111	India	Cross-sectional study; Questionnaire consisting of 18 closed-ended questions	83.8% of dental practitioners follow BMW disposal policies, 91% use protective gear, 63.1% properly dispose of anatomical and blood waste, 55.9% use yellow bags for teeth/tissue, and both general dentists and orthodontists correctly use needle destroyers for sharps (*p* = 0.041).	Between 7% and 9.2% of dentists do not adhere to established BMW management practices, and 9% do not use appropriate protective barriers during disposal. Increasing awareness of BMW management could be addressed through targeted camps and training sessions.
Nityae et al., 2024 [59]	Students (final year, interns, and postgraduates)	100	India	Cross-sectional study, Questionnaire with 28 items	The score of overall knowledge, attitude, and practice for the students in the final year of study, interns, and postgraduates was 67.5%, 68.09% and 71.76%, respectively.	Implementation of proper waste segregation, and using colour coding, safe storage and controlled transportation and disposal are mandatory for all dentists.
Pawar et al., 2024 [60]	Dentists, general physicians and nurses	150	India	Cross-sectional study Structured questionnaire with 18 items	Less than half of healthcare staff knew the correct biomedical waste storage time, and only 34% had excellent management knowledge. General physicians were most informed, then nurses and dentists. For sharp waste, most dentists and physicians (70%) and 60% of nurses correctly identified white or blue containers.	The study finds that ongoing training in BMW management is necessary for healthcare professionals, with targeted interventions needed due to varying knowledge and attitudes among dentists, physicians, and nurses.
Reshma et al., 2022 [61]	Dental undergraduate students (third year and final year) and house surgeons.	125	India	Cross-sectional study; Self-administered questionnaire with 45 questions	Most third-year participants and house surgeons knew BMW management, but 77.4% had no prior training. Few identified colour-coded bins correctly, and 30.4% used transparent containers for sharps.	The findings indicate a clear necessity for a comprehensive and continuous educational programme addressing the medical risks associated with inadequate waste management throughout all stages of the dental curriculum.
Revankar et al., 2023 [62]	Dental students	180	India	Cross-sectional study; Questionnaire with 21 items	70.4% of subjects provided correct answers; 74.37% were aware of medical waste management; there were statistically significant differences (*p* < 0.05) between correct and incorrect answers.	The students had a high level of awareness regarding medical waste management.
Sabbahi et al., 2020 [63]	General dentists, interns and specialists	306	Jeddah, Saudi Arabia	Cross-sectional study; Self-administered, close-ended online questionnaire with 18 items	Only 20% knew how to manage dental waste; 33% had training, and 73.2% understood waste coding. Knowledge and behaviour were significantly related to specialty, practice type, experience, and positively correlated (rs = 0.379, *p* < 0.001).	The participants had a low level of knowledge regarding the separation, collection, transport, and treatment of dental waste. Correct answers did not exceed the 20% threshold.
Sajid et al., 2025 [64]	Dental students of third year, final year students and Interns from 26 dental colleges	387	India, Kerala	Cross-sectional study; A 12-item, self-administrated, structured questionnaire	74.4% of fourth-year students obtained the lowest knowledge score (1–4 correct answers) regarding BMW management; 59% of interns had a low level of knowledge. Statistically significant differences (*p* = 0.000) were observed only regarding gender distribution, with male students showing a higher level of knowledge than female students.	The authors concluded that 69.3% of students had a low level of knowledge, while the proportion of those with a high level (9–12 correct answers) was only 2.1%. Third-year students had the highest knowledge scores compared to fifth-year students or interns.
Sarvathikari et al., 2022 [65]	Dental practitioners	54	India	Cross-sectional study; Questionnaire with 13 items	There were practitioners having no knowledge about BMW management and who did not practice colour-coded bins segregation. A quarter of dental practitioners declared they have difficulties in BMW management (too large bins, low frequency of waste collection, additional expenses).	Despite the awareness of dental practitioners about BMW management importance, there is a lack of knowledge and a lot of challenges in rules application.
Subramanian et al., 2020 [66]	Dentists (students, academicians, and clinicians)	355	India	Cross-sectional study; Questionnaire with 23 items	Increased percentage of the students, practitioners, and academicians (81.6%, 88.7%, and 76.9%, respectively) were aware of the laws and rules regarding BMW management. Low level of awareness was observed regarding the management of sharp wastes, amalgam, chemical waste and incineration procedure.	Although most participants knew the laws and rules regarding BMW management and the methods of segregation, the practice is not satisfactory.
Tatiya and Dandekeri, 2022 [67]	Undergraduate students, postgraduate students, and practitioners	162	India	Cross-sectional study; Questionnaire with 23 items	Despite an increased percentage (87.7%) of the participants being aware of BMW management according to new regulations, there was a gap between the knowledge and the implementation of waste segregation and disposal.	A certified carrier service should verify the segregated biomedical materials resulting from dental clinics and laboratories. Frequent updates of BMW management should be performed by the certified carrier.

## Data Availability

The data that support the findings of this study are available on request from the corresponding author.

## Supplementary Materials

The following supporting information can be downloaded at: https://www.mdpi.com/article/10.3390/dj13120594/s1, Table S1: PRISMA-ScR Checklist.

## Abbreviations

The following abbreviations are used in this manuscript:

BMW	Biomedical waste
FDI	World Dental Federation
WHO	World Health Organization

## References

[1] Hackley D.M., Luca J. (2024). Sustainability in Dentistry: An Overview for Oral Healthcare Team Members. J. Calif. Dent. Assoc..

[2] Batsford H., Shah S., Wilson G.J. (2022). A Changing Climate and the Dental Profession. Br. Dent. J..

[3] World Health Organization Climate Change. WHO Fact Sheets 2021. https://www.who.int/news-room/fact-sheets/detail/climate-change-and-health.

[4] Künzle P., Frank A.C., Paris S. (2025). Environmental Impact of a Tooth Extraction: Life Cycle Analysis in a University Hospital Setting. Community Dent. Oral Epidemiol..

[5] Sijm-Eeken M., Jaspers M., Peute L. (2023). Identifying Environmental Impact Factors for Sustainable Healthcare: A Scoping Review. Int. J. Environ. Res. Public Health.

[6] Nassar M., Shalan W., Al-Janaby U., Elnagar H., Alawadhi M., Jaser S., Joury E. (2024). Exploring Environmental Sustainability in Dentistry among Students and Educators in the United Arab Emirates: A Cross-Sectional Survey. BMC Med. Educ..

[7] Khurshid Z., Alqurashi H., Ashi H. (2024). Advancing Environmental Sustainability in Dentistry and Oral Health. Eur. J. Gen. Dent..

[8] Al Shatrat S.M., Shuman D., Darby M.L., Jeng H.A. (2013). Jordanian Dentists’ Knowledge and Implementation of Eco-Friendly Dental Office Strategies. Int. Dent. J..

[9] Martin N., Mulligan S. (2022). Environmental Sustainability through Good-Quality Oral Healthcare. Int. Dent. J..

[10] Spaveras A., Antoniadou M. (2023). Awareness of Students and Dentists on Sustainability Issues, Safety of Use and Disposal of Dental Amalgam. Dent. J..

[11] Khanna S.S., Dhaimade P.A. (2019). Green Dentistry: A Systematic Review of Ecological Dental Practices. Environ. Dev. Sustain..

[12] Macrì M., D’Albis V., Marciani R., Nardella M., Festa F. (2024). Towards Sustainable Orthodontics: Environmental Implications and Strategies for Clear Aligner Therapy. Materials.

[13] Mazur M., Ndokaj A., Jedlinski M., Maruotti A., Stamegna C., Corridore D., Capocci M., Ottolenghi L., Guerra F. (2019). How Dentistry Is Impacting the Environment. Senses Sci..

[14] Mittal R., Maheshwari R., Tripathi S., Pandey S. (2020). Eco-Friendly Dentistry: Preventing Pollution to Promoting Sustainability. Indian J. Dent. Sci..

[15] Sant I., Tripathi P., Chandra S., Sinha S. (2025). Eco-Dentistry: Sustainable Practices for Healthier Life and a Greener Planet. Asian J. Oral Health Allied Sci..

[16] Liu C.M., Yu C.H., Chang Y.C. (2023). Current Eco-Friendly Dentistry to Enhance Environmental Sustainability in Taiwan. J. Dent. Sci..

[17] Satchanska G., Davidova S., Petrov P.D. (2024). Natural and Synthetic Polymers for Biomedical and Environmental Applications. Polymers.

[18] Bamedhaf O., Salman H., Tegginmani S.A., Guraya S.S. (2025). Environmental Sustainability in the Dental Curriculum: A Scoping Review. BMC Med. Educ..

[19] Attrah M., Elmanadely A., Akter D., Rene E.R. (2022). A Review on Medical Waste Management: Treatment, Recycling, and Disposal Options. Environments.

[20] Yeoh S., Bourdamis Y., Saker A., Marano N., Maundrell L., Ramamurthy P., Sharma D. (2024). An Investigation into Contaminated Waste Composition in a University Dental Clinic: Opportunities for Sustainability in Dentistry. Clin. Exp. Dent. Res..

[21] Wadhawan R., Mishra S., Parihar S., Raj N., Rajput B., Kumar S., Devi L.M., Manauwwar M. (2025). Eco-Friendly Dentistry: Understanding the Environmental Impact in Dental Practice. J. Dent. Spec..

[22] World Health Organization Health-Care Waste. WHO Fact Sheets 2024. https://www.who.int/news-room/fact-sheets/detail/health-care-waste.

[23] Rodrigues de Sousa A.T., Pataca L.C.M., Maia C.C., Vimieiro G.V., Gonçalves M.F., Gomes Mol M.P. (2024). Waste Management from Dental Clinics: A Case Study in Belo Horizonte, Brazil. Waste Manag..

[24] Antoniadou M., Varzakas T., Tzoutzas I. (2021). Circular Economy in Conjunction with Treatment Methodologies in the Biomedical and Dental Waste Sectors. Circ. Econ. Sustain..

[25] Ozbek M., Sanin F.D. (2004). A Study of the Dental Solid Waste Produced in a School of Dentistry in Turkey. Waste Manag..

[26] Mandalidis A., Topalidis A., Voudrias E.A., Iosifidis N. (2018). Composition, Production Rate and Characterization of Greek Dental Solid Waste. Waste Manag..

[27] Wilmott S., Duane B. (2023). An Update on Waste Disposal in Dentistry. Br. Dent. J..

[28] Chanioti M., Nikolelis G., Mitsika I., Antoniadou M. (2025). The Role of Dentists in Promoting Environmental Awareness and Climate Consciousness for Sustainability. Circ. Econ. Sustain..

[29] Mitsika I., Chanioti M., Antoniadou M. (2024). Dental Solid Waste Analysis: A Scoping Review and Research Model Proposal. Appl. Sci..

[30] Khanna R., Konyukhov Y., Maslennikov N., Kolesnikov E., Burmistrov I. (2023). An Overview of Dental Solid Waste Management and Associated Environmental Impacts: A Materials Perspective. Sustainability.

[31] World Dental Federation (2018). Sustainability in Dentistry: Adopted by the FDI General Assembly: August 2017, Madrid, Spain. Int. Dent. J..

[32] Husaini D.C., Bernardez V., Zetina N., Mphuthi D.D. (2024). Healthcare industry waste and public health: A systematic review. Arab. Gulf J. Sci. Res..

[33] Jamal H., Marghalani A.A., Al-Sharif A., Shinawi A., Gaffar B., Al-Edaili E.A., Al-Baqami G., AlQarni M. (2023). Exploring the Perception of Dental Undergraduate Students and Faculty on Environmental Sustainability in Dentistry: A Cross-Sectional Survey in 26 Dental Schools in Saudi Arabia. Dent. J..

[34] Joury E., Lee J., Parchure A., Mortimer F., Park S., Pine C., Ramasubbu D., Hillman L. (2021). Exploring Environmental Sustainability in UK and US Dental Curricula and Related Barriers and Enablers: A Cross-Sectional Survey in Two Dental Schools. Br. Dent. J..

[35] Al-Thunian F.F., Al-Bounni R.S., Ingle N.A., Assery M.K. (2020). Evaluation of Green Dental Practice Implementation among Dental Practitioners Worldwide-A Systematic Review. J. Dent. Oral Health.

[36] Feres M., Albuini M., de Araújo Castro Santos R., de Almeida-Junior L.A., Flores-Mir C., Roscoe M.G. (2022). Dentists’ Awareness and Knowledge of Evidence-Based Dentistry Principles, Methods and Practices: A Systematic Review. Evid. Based Dent..

[37] Rai R., Krupetsky R., Howard S., Banava S. (2025). Innovating for Impact: Student Leadership in Sustainable Dental School Waste Management. J. Dent. Educ..

[38] Aromataris E., Lockwood C., Porritt K., Pilla B., Jordan Z. (2024). JBI Manual for Evidence Synthesis.

[39] Tricco A.C., Lillie E., Zarin W., O’Brien K.K., Colquhoun H., Levac D., Moher D., Peters M.D.J., Horsley T., Weeks L. (2018). PRISMA Extension for Scoping Reviews (PRISMA-ScR): Checklist and Explanation. Ann. Intern. Med..

[40] Daudt H.M., Van Mossel C., Scott S.J. (2013). Enhancing the Scoping Study Methodology: A Large, Inter-Professional Team’s Experience with Arksey and O’Malley’s Framework. BMC Med. Res. Methodol..

[41] Ansari S.H., Al Neemi N.A., Al Jadaan S., Alsenan F.K., Aldawsari F.S., Saleh F.K. (2020). The Disposal of Biomedical Waste by the Dental Health Professionals in Riyadh: Impact on Current Practice. Med. Sci..

[42] Bawa R., Khurana D., Girdhar P., Verma N. (2022). Biomedical Waste Management-Related Knowledge, Attitude, and Practices among Clinical Dental Undergraduates in the State of Punjab, India: A Cross-Sectional Study. Dent. J. Adv. Stud..

[43] Boukhris H., Zidani H., Khalifa A.B.H., Bouslema G., Youssef S.B. (2025). Environmental Impact of Dental Waste: A Survey-Based Analysis of Waste Segregation and Recycling Practices in Dental Clinics. J. Contemp. Dent. Pract..

[44] Cayo-Rojas C., Briceño-Vergel G., Córdova-Limaylla N., Huamani-Echaccaya J., Castro-Mena M., Lurita-Córdova P., Bermúdez-Mendoza J., Allen-Revoredo C., Torres-Vásquez J., Ladera-Castañeda M. (2023). Impact of a Virtual Educational Intervention on Knowledge and Awareness of Biomedical Waste Management among Peruvian Dental Professionals. Sci. Rep..

[45] Choudhary M., Verma M., Ghosh S., Dhillon J.K. (2020). Assessment of Knowledge and Awareness about Biomedical Waste Management among Health Care Personnel in a Tertiary Care Dental Facility in Delhi. Indian J. Dent. Res..

[46] Diaz-Soriano A., Gallo W., Luza S., Munive-Degregori A., Bocanegra R., Mayta-Tovalino F. (2020). Knowledge and Awareness of Effective Recycling of Dental Materials and Waste Management among Peruvian Undergraduate Students of Dentistry: A Logistic Regression Analysis. J. Int. Soc. Prev. Community Dent..

[47] Gao Y., Huang X., Jin Z., Zhang X., Liu Y., Chen J. (2025). Tailored Role-Specific Training Program Improves Knowledge, Attitudes, and Practices in Medical Waste Management among Dental Professionals. Sci. Rep..

[48] Ghanem E.A., Elhossiney D.M., Gamal D.A. (2025). Biomedical Waste Management, Mercury Hygiene Practices and Associated Factors among Dentists and Dental Students. Egypt. J. Occup. Med..

[49] Gowdar I.M., Al-Mansour O.A., Alshehri M.A., Alaskar A.M., Alfahad M.H., Al-Harbi K.F. (2024). Biomedical Waste Management Knowledge among Dental Students and Private Dental Practitioners of Alkharj, Saudi Arabia. J. Pharm. Bioallied Sci..

[50] Kamran M.A., Zareef U., Ahmed T., Rasool R., Khan N., Kashif M. (2022). Awareness of Dental Undergraduates, Postgraduates and Dental Practitioners about Dental and Biomedical Waste Management. J. Liaquat Univ. Med. Health Sci..

[51] Khubchandani K., Devi K.M., Gunasekaran S., Yeturu S.K., Ramanarayanan V. (2020). Knowledge, Attitude, and Practices of Biomedical Waste Management among Clinical Dental Students. J. Glob. Oral Health.

[52] Lakbala P. (2020). Dental Waste Management among Dentists of Bandar Abbas, Iran. AIMS Environ. Sci..

[53] Lakhani B., Givati A. (2024). Perceptions and Decision-Making of Dental Professionals to Adopting Sustainable Waste Management Behaviour: A Theory of Planned Behaviour Analysis. Br. Dent. J..

[54] Mahajan A., Pawar M., Patil A.N., Behera S., Pattnaik S.J., Rajguru J.P. (2024). Biomedical Waste Management: A Study on the Awareness and Practice among Dental Healthcare Workers in India. J. Int. Clin. Dent. Res. Organ..

[55] Mahesh S., Hemalata K., Shanta R., Vashistha U., Krishnakumar K., Arora S., Gupta A. (2025). Bridging the Gap: A Cross-Sectional Study on Biomedical Waste Management Education and Compliance in Dental Institutions of Delhi National Capital Region. GMS Hyg. Infect. Control.

[56] Mayta-Tovalino F., Munive-Degregori A., Bocanegra R., Alvitez J., Temoche A. (2022). Awareness, Knowledge, Attitude, and Practices in the Management of Biomedical Waste: A Multivariate Analysis of Associated Factors in Peruvian Students. World J. Dent..

[57] Makanjuola J.O., Ekowmenhenhen U.I., Enone L.L., Umesi D.C., Ogundana O.M., Arotiba G.T. (2021). Mercury Hygiene and Biomedical Waste Management Practices among Dental Health-Care Personnel in Public Hospitals in Lagos State, Nigeria. Afr. Health Sci..

[58] Monica K., Abilasha R., Ramani P., Gheena S., Reshma P.K. (2022). Knowledge and Awareness on Management of Biomedical Waste among Orthodontists and General Dental Practitioners. Int. J. Orthod. Rehabil..

[59] Nitya K., Perumal P., Kumarasamy B., Gangadharamurthy B. (2024). From Classroom to Clinic: A Cross-Sectional Survey on Fostering Awareness of Biomedical Waste Management among Dental Students. SRM J. Res. Dent. Sci..

[60] Pawar M.D., Kamala K.A., Pawar M. (2024). Knowledge, Awareness, and Perceived Attitude of Biomedical Waste Management among Healthcare Personnel. Cureus.

[61] Reshma S., Arshiya S., Prabhath E.K., Sekhar P.R., Ranganath S. (2022). Awareness on Biomedical Waste Management among Dental Students: A Cross-Sectional Questionnaire Survey. Int. J. Adv. Res..

[62] Revankar V.D., Ponnusamy C., Subramanian A., Noon A.M., Subhashini M., Saravanaraja M. (2023). Knowledge of Biomedical Waste Management Amidst the Clinical Students of Dental College, Tamil Nadu State, India—A Cross-Sectional Observational Study. J. Pharm. Bioallied Sci..

[63] Sabbahi D.A., El-Naggar H.M., Zahran M.H. (2020). Management of Dental Waste in Dental Offices and Clinics in Jeddah, Saudi Arabia. J. Air Waste Manag. Assoc..

[64] Sajid S., George B., Soman R.R. (2025). Knowledge on Biomedical Waste Management Systems in Kerala among Dental Students. Clin. Dent..

[65] Sarvathikari R., Pavithran V.K., Ravichandiran R. (2021). Challenges in Implementation of Biomedical Waste Management among the Dental Practitioners in a Tier 2 Town in India-A Cross-Sectional Study. Indian J. Dent. Res..

[66] Subramanian A.K., Nivethigaa B., Srirengalakshmi M., Varghese R.M., Navaneethan R., Babu H. (2020). Biomedical Waste Management Practice in Dentistry. Bioinformation.

[67] Tatiya V., Dandekeri S. (2023). Knowledge Assessment of Biomedical Waste Management of Dental Materials in Dakshina Kannada. J. Health Allied Sci. NU.

[68] Guerra M., Morgado M., Leira Y., Leitão T., Botelho J., Mendes J.J. (2025). Integrating Sustainability in Dentistry: A Pathway towards Achieving the UN 2030 Agenda. Front. Oral Health.

[69] Sakchhi S., Elbanna L., Chakor M., Nikferjam A.Z., Saeedi N., Badve S., Singh S. (2025). Green Dentistry: Sustainable Practices and Materials for a Healthier Planet. Int. J. Dent. Mater..

[70] Zia N., Doss J.G., John J., Panezai J. (2024). Sustainability in Dentistry: Assessing Knowledge, Attitude, and Practices of Dental Practitioners about Green Dentistry. Pak. J. Med. Sci..

[71] Speroni S., Polizzi E. (2025). Green Dentistry: State of the Art and Possible Development Proposals. Dent. J..

[72] Țâncu A.M.C., Imre M., Iosif L., Pițuru S.M., Pantea M., Sfeatcu R., Ilinca R., Bodnar D.C., Didilescu A.C. (2025). Is Sustainability Part of the Drill? Examining Knowledge and Awareness among Dental Students in Bucharest, Romania. Dent. J..

[73] Wolf T.G., Campus G. (2021). Changing Dental Profession—Modern Forms and Challenges in Dental Practice. Int. J. Environ. Res. Public Health.

[74] Hsu L.P., Huang Y.K., Chang Y.C. (2022). The Implementation of Artificial Intelligence in Dentistry Could Enhance Environmental Sustainability. J. Dent. Sci..

[75] Dormen M., Özkan P. (2025). Sustainable Dental Approaches for the Environment and Human Health: A Traditional Literature Review. HRU Int. J. Dent. Oral Res..

[76] Duane B., Dixon J., Ambibola G., Aldana C., Coughlan J., Henao D., Daniela T., Veiga N., Martin N., Darragh J. (2021). Embedding Environmental Sustainability within the Modern Dental Curriculum: Exploring Current Practice and Developing a Shared Understanding. Eur. J. Dent. Educ..

[77] Haque S., Nurunnabi M., Haque T. (2024). Saudi Dental Students’ Perceptions on Sustainable Development Goals and Sustainable Dental Practice. BDJ Open.

[78] Durnall O., Martin N., Mulligan S., Dixon J. (2024). Environmental Sustainability: The Attitudes and Experiences of UK Students in the Oral Health Care Profession. Br. Dent. J..

[79] Hassan E.H., Lotfy N., Abdou M.H., Fetohy E.M., Hussein M.F. (2025). Effectiveness of an Environmental Educational Program on Intern Dentists’ Knowledge and Practices Regarding Eco-Friendly Green Dentistry: A Quasi-Experimental Study. BMC Med. Educ..

[80] Bano V., Amin E., Maqbool S., Hassan S.A., Baber A., Urooj R. (2024). Awareness of Green Dentistry Concept among Dental Professionals. Life Sci..

[81] Dixon J., Field J., Gibson E., Martin N. (2024). Curriculum Content for Environmental Sustainability in Dentistry. J. Dent..

[82] Al-Rabab’ah M.A., Bustani M.A., Khraisat A.S., Sawair F.A. (2016). Phase Down of Amalgam: Awareness of Minamata Convention among Jordanian Dentists. Saudi Med. J..

[83] Mackey T.K., Contreras J.T., Liang B.A. (2014). The Minamata Convention on Mercury: Attempting to Address the Global Controversy of Dental Amalgam Use and Mercury Waste Disposal. Sci. Total Environ..

[84] Eshrati M., Momeniha F., Momeni N., Ahmadi E., Hashemian A., Kashani H., Alaeddini M. (2024). Clinical Guide Adaptation for Amalgam Waste Management in Dental Settings in Iran. Front. Dent..

[85] European Union (2024). Regulation (EU) 2024/1849 of the European Parliament and of the Council. Off. J. Eur. Union.

[86] Disha S. Dental Amalgam Set to Be Phased Out by 2034 to Reduce Toxic Mercury Exposures. Health Policy Watch, Health & Environment 2025. https://healthpolicy-watch.news/dental-amalgam-set-to-be-phased-out-by-2034-to-reduce-toxic-mercury-exposures/.

[87] Tibau A.V., Grube B.D. (2019). Mercury Contamination from Dental Amalgam. J. Health Pollut..

[88] Environment Agency Healthcare Waste: Appropriate Measures for Permitted Facilities. Gov.UK 2021. https://www.gov.uk/guidance/healthcare-waste-appropriate-measures-for-permitted-facilities/waste-storage-segregation-and-handling-appropriate-measures.

[89] Gao Q., Shi Y., Mo D., Nie J., Yang M., Rozelle S., Sylvia S. (2018). Medical waste management in three areas of rural China. PLoS ONE.

[90] De Leon M.L. (2020). Barriers to Environmentally Sustainable Initiatives in Oral Health Care Clinical Settings. Can. J. Dent. Hyg..

[91] Martin N., Sheppard M., Gorasia G., Arora P., Cooper M., Mulligan S. (2021). Awareness and Barriers to Sustainability in Dentistry: A Scoping Review. J. Dent..

